# Força de Preensão Manual na Insuficiência Cardíaca: Construção de uma Equação de Referência

**DOI:** 10.36660/abc.20240777

**Published:** 2025-10-13

**Authors:** Suena Medeiros Parahiba, Édina Caroline Ternus Ribeiro, Ingrid da Silveira Knobloch, Débowra Dapper, Ingrid Dalira Schweigert Perry, Nadine Oliveira Clausell, Vivian Luft, Gabriela Corrêa Souza, Eneida Rejane Rabelo-Silva

**Affiliations:** 1 Universidade Federal do Rio Grande do Sul Faculdade de Medicina Porto Alegre RS Brasil Programa de Pós-Graduação em Ciências da Saúde, Cardiologia e Ciências Cardiovasculares, Faculdade de Medicina, Universidade Federal do Rio Grande do Sul, Porto Alegre, RS – Brasil; 2 Universidade Federal de Ciências da Saúde de Porto Alegre Porto Alegre RS Brasil Universidade Federal de Ciências da Saúde de Porto Alegre, Porto Alegre, RS – Brasil; 3 Hospital de Clínicas de Porto Alegre Porto Alegre RS Brasil Hospital de Clínicas de Porto Alegre, Porto Alegre, RS – Brasil; 4 Universidade Federal do Rio Grande do Sul Faculdade de Medicina Porto Alegre RS Brasil Programa de Pós-Graduação em Alimentação, Nutrição e Saúde, Faculdade de Medicina, Universidade Federal do Rio Grande do Sul, Porto Alegre, RS – Brasil; 5 Universidade Federal do Rio Grande do Sul Escola de Enfermagem Porto Alegre RS Brasil Escola de Enfermagem, Universidade Federal do Rio Grande do Sul, Porto Alegre, RS – Brasil

**Keywords:** Insuficiência Cardíaca, Força da Mão, Valores de Referência

## Abstract

**Fundamento:**

A força de preensão manual (FPM) é um indicador fundamental da força muscular global e da capacidade funcional em pacientes com insuficiência cardíaca (IC). No entanto, ainda não foram publicadas equações de referência específicas para essa população.

**Objetivo:**

Este estudo teve como objetivo desenvolver e validar uma equação preditiva da FPM em pacientes com IC.

**Métodos:**

Foi realizado um estudo transversal com pacientes com IC estável, com idade entre 18 e 79 anos, diagnosticados há pelo menos três meses. O valor máximo da FPM foi obtido a partir de três medições consecutivas. Dados clínicos e avaliações antropométricas foram coletados. A amostra foi dividida aleatoriamente em dois terços (n=174) para derivação e um terço (n=100) para validação. Um modelo de regressão multivariada foi aplicado para desenvolver a equação preditiva, incluindo variáveis com valor de p < 0,25 conforme determinado pelo teste de Wald.

**Resultados:**

As amostras de derivação e validação não apresentaram diferenças significativas nas características basais. Os pacientes eram predominantemente homens, idosos e brancos. A equação preditiva derivada foi: FPM prevista = −39,732 + (10,771 × sexo [feminino = 0; masculino = 1]) − (0,158 × idade [anos]) + (35,096 × altura [m]) + (0,448 × circunferência da panturrilha [cm]) − (4,224 × classe da NYHA [I/II = 0; III/IV = 1]). Ao ser aplicada na amostra de validação, a equação subestimou a FPM real em 0,68 ± 8,93 kg.

**Conclusão:**

Idade, sexo, altura, circunferência da panturrilha e classe NYHA foram os principais determinantes da FPM em pacientes com IC. A equação derivada apresentou boa acurácia preditiva e pode servir como referência útil para interpretar medições de força de preensão nessa população.

## Graphical abstract

**Figura Central:**
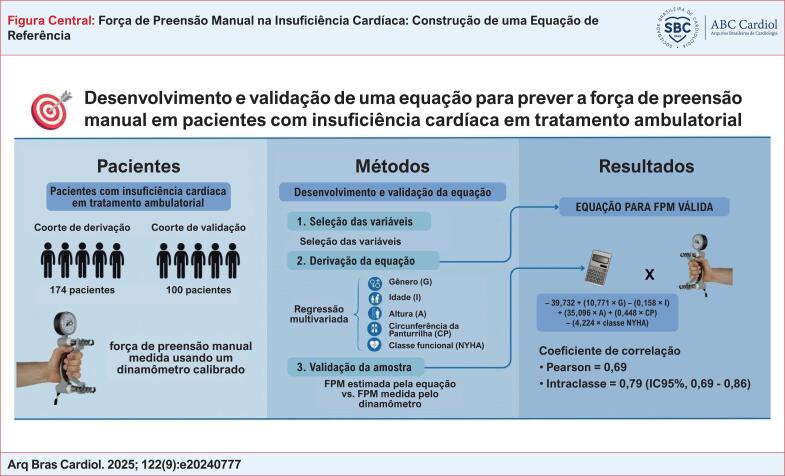
Força de Preensão Manual na Insuficiência Cardíaca: Construção de uma Equação de Referência

A equação proposta mostrou boa concordância em predizer a força de preensão manual (FPM) e pode auxiliar na interpretação da medida em pacientes com insuficiência cardíaca (IC).

## Introdução

A capacidade funcional oferece uma avaliação direta e não invasiva do estado físico e está associada ao prognóstico e a outros desfechos clínicos.^1^ A força de preensão manual (FPM) está relacionada à fragilidade, uma condição que afeta cerca de 40% dos pacientes com insuficiência cardíaca (IC). A fragilidade está intimamente ligada ao envelhecimento, ao declínio cognitivo e ao aumento da complexidade no manejo clínico dessa população.^2 – 5^

A FPM tem sido investigada como uma alternativa simples e de baixo custo para avaliar a capacidade funcional, podendo ser facilmente aplicada em ambientes clínicos.^5 , 6^ É uma medida influenciada por fatores antropométricos, sexo e envelhecimento. Embora a FPM avalie principalmente um grupo de músculos do membro superior, ela é considerada um indicador da força muscular global.^7 – 9^

Recentemente reconhecida como um parâmetro prognóstico em pacientes com IC, a FPM foi avaliada em uma revisão sistemática que incluiu 7350 pacientes com períodos de acompanhamento variando de três a 43 meses.^6^ Foi demonstrado que uma diminuição em 1 Kg na FPM está associada a um aumento de 8% no risco de mortalidade (risco relativo de 1,08; intervalo de confiança – IC – de 95%: 1,05 a 1,11; p < 0,001).

Diante das evidências apresentadas, a FPM pode se tornar um marcador de rotina para a avaliação e monitoramento de pacientes com IC na prática clínica.^5 , 10 , 11^ O uso de equações e tabelas de referência representa um método alternativo de predição e avaliação desse parâmetro. No entanto, os dados disponíveis na literatura são baseados em populações saudáveis, e as fórmulas atuais de predição da FPM não são adaptadas a condições clínicas que afetam a hemodinâmica, o que pode levar a avaliações imprecisas.^12 , 13^ Portanto, este estudo teve como objetivo desenvolver e validar uma equação de referência para a avaliação da FPM em pacientes com IC. A metodologia central do estudo está ilustrada na Figura Central .

## Métodos

### Seleção dos pacientes e dados clínicos

Este estudo transversal incluiu pacientes adultos e idosos (com idade entre 18 e 79 anos) com diagnóstico confirmado de IC, independentemente da classificação da fração de ejeção. Os participantes em atendimento ambulatorial foram recrutados por conveniência na clínica de IC de um hospital público de alta complexidade no Brasil. A coleta de dados foi realizada entre agosto de 2018 e julho de 2023.

Foram excluídos pacientes com histórico de transplante cardíaco; IC descompensada aguda; infarto agudo do miocárdio, acidente vascular cerebral ou cirurgia nos três meses anteriores à participação no estudo; congestão periférica e/ou edema; histórico de angina instável; em terapia renal substitutiva; com tumores malignos (ativos ou em remissão) nos últimos cinco anos; infecção aguda; diagnóstico prévio de doença neurodegenerativa; ou incapacidade de realizar testes funcionais (por exemplo, usuários de cadeira de rodas, amputados, indivíduos com sequelas motoras ou deficiências). Critérios adicionais de exclusão incluíram contraindicações para análise de bioimpedância elétrica, como presença de próteses metálicas e índice de massa corporal (IMC) superior a 39 Kg/m^2^.

A etiologia da IC, a classificação funcional (segundo a *New York Heart Association* – NYHA), o tratamento farmacológico, as comorbidades e outros dados clínicos e sociodemográficos foram obtidos a partir de registros médicos eletrônicos e confirmados durante a anamnese realizada por pesquisadores treinados. Os dados incluíram avaliação antropométrica de peso (Kg), altura (m), IMC (Kg/m^2^), circunferência do braço (cm), prega cutânea tricipital (mm), circunferência muscular do braço (cm) e circunferência da panturrilha (cm). As avaliações foram realizadas por pesquisadores treinados, certificados por um supervisor experiente antes do início da coleta de dados.

### Força de preensão manual

A FPM foi avaliada utilizando um dinamômetro manual Jamar calibrado (Sammons Preston, Bolingbrook, IL, EUA). As avaliações foram realizadas com a mão dominante, com os pacientes sentados, quadris flexionados a 90°, braços ao lado do tronco e cotovelos flexionados a 90°.^14 , 15^ Os pacientes foram instruídos a apertar o aparelho com força máxima e receberam incentivo verbal do pesquisador.^16^ Foram realizadas três contrações máximas, com intervalo de um minuto entre cada tentativa. O maior valor entre as três medições foi registrado como resultado final da HGS.

A força de preensão manual prevista (FPMp) foi calculada utilizando uma equação de desenvolvida para pacientes com IC, conforme detalhado na seção de "análise estatística".

### Aspectos éticos

Este estudo foi aprovado por um Comitê de Ética em Pesquisa (protocolo número 5057.8121.200005327), e todos os pacientes assinaram um termo de consentimento livre e esclarecido.

### Análise estatística

A análise estatística foi realizada utilizando o SPSS ( *Statistical Package for the Social Sciences* ), versão 18.0 (IBM Corp., Armonk, NY, EUA). As variáveis contínuas foram expressas como média e desvio padrão ou mediana e intervalo interquartil, e as variáveis categóricas foram expressas como valores absolutos e relativos. A normalidade das variáveis contínuas foi avaliada por meio do teste de Kolmogorov-Smirnov.

Após a coleta dos dados, dois terços dos participantes foram alocados aleatoriamente para formar a amostra de derivação utilizando o comando de seleção de casos no SPSS. Os participantes restantes foram incluídos na coorte de validação. Para avaliar a similaridade entre as amostras de derivação e validação, foram realizadas comparações utilizando o teste do qui-quadrado de Pearson (χ^2^) para variáveis categóricas, e o teste t de Student não pareado ou o teste de Mann-Whitney para variáveis contínuas, conforme apropriado. Um nível de significância de p = 0,05 foi adotado para todas as comparações.

Para desenvolver a equação preditiva na amostra de derivação, foi realizada inicialmente uma análise de regressão univariada. Variáveis com valor de p < 0,25 (teste de Wald) foram selecionadas para inclusão no modelo de regressão multivariada subsequente e para avaliação do coeficiente de correlação.^17 – 19^

Três modelos preditivos foram desenvolvidos para estimar a FPM em pacientes com IC (Material Suplementar). O modelo final foi selecionado com base em seu desempenho estatístico e adequação às características da população estudada. Subsequentemente, a equação foi aplicada à amostra de validação para calcular os valores de FPMp. A acurácia preditiva foi avaliada por meio da análise de resíduos e correlação. Os resíduos foram calculados subtraindo os valores preditos dos valores observados: Resíduos = FPM observada (medida por dinamometria) – FPMp (derivada da equação de referência).

A concordância entre os valores observados e os preditos foi avaliada utilizando o coeficiente de correlação intraclasse (ICC), que quantifica a concordância absoluta. Valores de ICC <0,5 foram considerados baixos, valores entre 0,5 e 0,75 moderados, valores entre 0,75 e 0,90 bons, e entre 0,90 e 1,0 considerados excelentes.^20^ Gráficos de dispersão foram utilizados para visualizar a correlação entre os valores observados e os preditos.

## Resultados

O estudo incluiu 274 pacientes com IC clinicamente estáveis, predominantemente homens idosos, autodeclarados como de cor branca, sendo 82% classificados como NYHA classe I ou II ( Tabela 1 ). A amostra de derivação (n = 174) e a amostra de validação (n = 100) não apresentaram diferenças significativas nas características demográficas ou clínicas.

**Tabela 1:** Características demográficas e clínicas de pacientes com insuficiência cardíaca nas amostras de derivação e validação, segundo faixa etária

Variável	Amostra de derivação (n = 174)	Amostra de validação (n = 100)	Valor p
Idade, anos	62,0 (53,8-67)	62,0 (57-67,8)	0,331
Idosos	104 (59,8)	62 (62)	0,716
Sexo masculino	111 (63,8)	74 (74)	0,082
Etnia branca	129 (74,6)	79 (79,0)	0,407
Fração de ejeção, %	31 (24-41)	32,5 (25-40)	0,842
NYHA (I/II)	142 (81,6)	84 (84)	0,616
Diabetes	52 (29,9)	31 (31,0)	0,847
HAS	91 (52,3)	48 (48,0)	0,493
DRC	14 (8,0)	11 (11,0)	0,414
Dislipidemia	17 (9,8)	8 (8,0)	0,624
DAC	13 (7,5)	9 (9,0)	0,654
FA	29 (16,7)	21 (21,0)	0,371
IECA/BRA ou sacubitril/valsartana	140 (80,0)	82 (81,2)	0,883
Betabloqueador	167 (96,5)	96 (96)	0,532
Diuréticos	144 (83,2)	87 (87,9)	0,303
Espironolactona	120 (70,6)	68 (69,4)	0,836
Peso, Kg	78,2 (64,5-89,5)	79,3 (65,6-90)	0,930
Altura, m	1,6±0,1	1,7±0,1	0,282
IMC, Kg/m²	28,5 (25-31,9)	28,6 (24,3-30,9)	0,722
CB, cm	32,7±4,1	32,4±4,2	0,609
PCT, mm	17,1 (13,1-23,9)	16,1 (12,6-21,7)	0,335
CMB, cm	26,7±2,9	26,8±3,7	0,898
CP, cm	38,1±3,9	37,9±3,7	0,744
FPM, Kg	28 (20-36,2)	30 (22-39,3)	0,176

CB: circunferência do braço; CMB: circunferência muscular do braço; CP: circunferência da panturrilha; DAC: doença arterial coronariana; DRC: doença renal crônica; FA: fibrilação atrial; FPM: força de preensão manual; FPM_*p*_: força de preensão manual predita; HAS: hipertensão arterial sistêmica; IC: insuficiência cardíaca; NYHA: classe funcional da insuficiência cardíaca congestiva segundo a New York Heart Association; PCT: prega cutânea tricipital; as variáveis contínuas são expressas como média e desvio padrão ou como mediana e intervalo interquartil, e as variáveis categóricas são expressas como valores absolutos e relativos.

Na análise da amostra de derivação (n = 174), as variáveis com maior poder explicativo para a FPM foram sexo, idade, altura, circunferência da panturrilha e classe funcional da NYHA, com valores de R^2^ ajustado de 0,427, 0,043, 0,446, 0,093 e 0,031, respectivamente. Com base nessas variáveis, a equação de predição para FPM em pacientes com IC (de 18 a 79 anos) foi: FPMp = −39,732 + (10,771 × sexo [feminino = 0; masculino = 1]) − (0,158 × idade [anos]) + (35,096 × altura [m]) + (0,448 × circunferência da panturrilha [cm]) − (4,224 × classe NYHA [I/II = 0; III/IV = 1]) ( Tabela 2 ). Esse modelo apresentou um R^2^ de 0,578 e um R^2^ ajustado de 0,565. Ao ser aplicado à amostra de derivação, o resíduo médio entre os valores observados e preditos foi de 0,025 ± 7,601 Kg, sendo considerado uma variável com comportamento simétrico (valor de p = 0,200).

**Tabela 2:** Coeficientes e estatísticas da análise de regressão multivariada para força de preensão manual (mão dominante) na amostra de derivação de pacientes com insuficiência cardíaca com idade entre 18 e 79 anos

Modelo	Coeficiente B não padronizado	IC95%	Valor p
Constante	-39,732	-70,070, -9,394	0,01
Sexo (feminino = 0; masculino = 1)	10,771	7,308, 14,233	<0,001
Idade (anos)	-0,158	-0,261, -0,054	0,003
Altura (m)	35,096	17,251, 52,941	0,000
Circunferência da panturrilha(cm)	0,448	0,138, 0,758	0,005
NYHA (I/II = 0; III/IV = 1)	-4,224	-7,234, -1,213	0,006

*IC: intervalo de confiança; NYHA: classe funcional da insuficiência cardíaca congestiva de acordo com a* New York Heart Association.

### Correlação

A equação foi aplicada à amostra de validação. Os diagramas de dispersão e suas respectivas linhas de ajuste linear estão apresentados na Figura Suplementar 1. O eixo y representa os valores observados da força de preensão (medidos com um dinamômetro) da mão dominante, enquanto o eixo x mostra os valores previstos (em Kg), derivados da equação de referência, também para a mão dominante. De modo geral, o coeficiente de correlação de Pearson (r) entre os valores observados e os previstos foi de 0,69, e o coeficiente de correlação intraclasse (ICC) foi de 0,79 (IC 95%: 0,69 a 0,86; p < 0,001), demonstrando boa concordância.

### Resíduos

Na amostra de validação, a FPMp média foi de 29,45 ± 8,90 kg, em comparação com uma força de preensão observada média de 30,13 ± 12,33 kg. Isso resultou em um resíduo médio de 0,68 ± 8,93 kg, indicando que a pHGS foi, em média, 680 g inferior ao valor observado.

## Discussão

Este estudo propõe uma equação de referência para prever os valores de FPM em pacientes com IC, com base em dados coletados de indivíduos clinicamente estáveis. Os dados foram obtidos seguindo protocolos previamente publicados na literatura.^14 , 15 , 17 – 19 , 21^ Os resultados indicam que 57% da variância na FPM pode ser explicada pela influência combinada de sexo, idade, estatura, classificação NYHA e circunferência da panturrilha. A equação proposta oferece uma alternativa para estimar os valores esperados de FPM em IC, permitindo que clínicos e pesquisadores comparem medidas em pacientes ambulatoriais e potencialmente extrapolem seu uso para situações de descompensação.

Apesar da forte correlação observada, os resíduos entre os valores preditos e os valores observados de FPM não podem ser ignorados. O mesmo foi descrito em um estudo populacional com indivíduos saudáveis, no qual a FPM foi predita com base na idade, estatura e peso.^2 2^ Nesse estudo, os resíduos médios foram de 1,41 ± 5,57 Kg para a mão dominante e 1,03 ± 5,44 Kg para a mão não dominante, respectivamente.^2 2^ Wang et al. também demonstraram que os valores de pFPM podem diferir dos valores medidos em uma proporção considerável de pacientes.^2 2^

Em nossa revisão de literatura anterior, não foram identificadas equações de referência que se aplicassem especificamente à população com IC. No entanto, grupos de pesquisa brasileiros desenvolveram equações de referência para indivíduos saudáveis da população brasileira em geral.^23 , 24^ Por exemplo, Novaes et al.^2^³ propuseram as seguintes equações: FPM (mão dominante) = 39,996 – [0,382 × idade (anos)] + [0,174 × peso (Kg)] + [13,628 × sexo (feminino = 0; masculino = 1)]; FPM (não dominante) = 44,968 – [0,420 × idade (anos)] + [0,110 × peso (Kg)] + [9,274 × sexo (feminino = 0; masculino = 1)].^23^

Esse estudo incluiu apenas indivíduos com 50 anos ou mais e relatou coeficientes de regressão ajustados de 0,677 para a mão dominante e 0,546 para a mão não dominante.^2^³ No entanto, ao serem testadas em uma população de homens brasileiros de meia-idade, os CCIs entre os valores preditos e medidos foram baixos — 0,52 para a mão dominante e 0,42 para a mão não dominante — indicando acurácia preditiva limitada.^25^ Esses resultados sugerem que as equações possuem validade restrita quando extrapoladas para populações diferentes daquelas originalmente estudadas.

Por outro lado, Lopes et al.^24^ desenvolveram equações preditivas para a FPM em indivíduos brasileiros saudáveis, adultos jovens e de meia-idade, incorporando variáveis adicionais como a circunferência do antebraço e o comprimento da mão, além do sexo. As equações propostas foram: FPM (mão dominante) = −15,490 + (10,787 × sexo [feminino = 0; masculino = 1]) + (0,558 × circunferência do antebraço [cm]) + (1,763 × comprimento da mão [cm]); e FPM (mão não dominante) = −9,887 + (12,832 × sexo [feminino = 0; masculino = 1]) + (2,028 × comprimento da mão [cm]). Essas equações foram capazes de explicar aproximadamente 71% da variabilidade na FPM.^24^

Uma análise das equações de referência revela que certas variáveis exercem maior influência sobre a FPM. Um estudo demonstrou que, entre as medidas antropométricas do antebraço e da mão, a largura da mão é o melhor preditor de FPM em adultos jovens.^26^ No entanto, a estatura é amplamente reconhecida na literatura como a variável antropométrica mais associada à FPM. A altura reflete parte da estrutura óssea, e a massa óssea também contribui para o desempenho muscular e de força.^9 , 27 – 29^ O *International Working Group on Sarcopenia* recomenda incluir a altura na avaliação da massa muscular relativa, especialmente no contexto da incapacidade funcional.^30^ Assim, a inclusão da estatura em modelos preditivos de FPM é essencial para obter valores condizentes com as características estruturais e anatômicas do indivíduo.

Como o esperado, houve diferença na força entre homens e mulheres, que se estendeu a outros grupos musculares do membro superior. Mulheres apresentam aproximadamente 30% da força masculina na flexão de ombro e na rotação interna/externa. Embora o mecanismo de força na escápula se mantenha consistente entre os padrões de movimento, as mulheres geram apenas entre 55% e 62% da força observada nos homens.^31^ Em relação à força do punho, as mulheres produzem aproximadamente 60–65% da força de flexão/extensão masculina, cerca de 55–60% da força de pronação/supinação e entre 60–70% da força de desvio ulnar/radial.^32^ Em geral, mulheres apresentam menor capacidade de produção de força em comparação aos homens; no entanto, quando a força é normalizada pela massa corporal, as diferenças entre os sexos tornam-se menos acentuadas – em alguns casos, as mulheres até superam os homens em métricas de força. Esse fenômeno é particularmente evidente durante movimentos de flexão e extensão em condições isocinéticas.^33^

A idade também desempenha papel significativo na predição da FPM, principalmente devido à redução da força muscular associada ao envelhecimento.^8^ Em humanos, os sistemas neuromuscular e sensorial são essenciais para a geração da força máxima de preensão.^33 , 34^ Com o passar dos anos, a degradação fisiológica desses sistemas pode levar à lentidão e à inconsistência na geração de força pelas mãos.

A equação preditiva para indivíduos com IC exigiu a inclusão da circunferência da panturrilha, um indicador de massa corporal magra. Essa decisão é respaldada por pesquisas anteriores que indicam que a FPM se correlaciona com a força muscular geral em pacientes.^7^ Além disso, a inclusão da classificação funcional da NYHA introduz uma variável clínica amplamente utilizada, baseada na avaliação das limitações da atividade física.^35^ Um estudo anterior demonstrou diferenças clinicamente significativas na FPM entre pacientes com maior comprometimento funcional, classificados como NYHA III/IV. Para cada quilograma adicional na FPM, houve uma redução de 2% na probabilidade de ser classificado como NYHA III ou IV.^36^

Equações de predição específicas para a FPM podem servir como ferramentas úteis para interpretação clínica, visto que os pacientes frequentemente apresentam valores basais significativamente abaixo dos padrões de referência estabelecidos para populações saudáveis. Para enfrentar as limitações decorrentes da ausência de valores de referência específicos para pacientes em hemodiálise, Dilloway et al.^37^ propuseram uma equação preditiva adaptada a esse grupo. A amostra de derivação foi composta por indivíduos bem nutridos, conforme a avaliação subjetiva global. Equações separadas foram desenvolvidas para cada sexo, utilizando variáveis demográficas como altura e idade. Por meio dessas equações, a FPM esperada foi calculada e comparada à FPM observada, medida por dinamometria. Essa comparação resultou no índice de FPM (%), que os autores sugeriram como um parâmetro personalizado que oferece uma avaliação mais precisa da fraqueza muscular em pacientes submetidos à hemodiálise.^37^

Em nosso conhecimento, este é o primeiro estudo a propor uma equação preditiva para a FPM em pacientes com IC. Embora o estudo apresente limitações, como a utilização de uma amostra de conveniência e a ausência de monitoramento longitudinal da FPM nos mesmos indivíduos, as fortes correlações e a alta acurácia preditiva da equação sugerem que ela pode se tornar uma ferramenta valiosa na prática clínica, especialmente para avaliação da fragilidade. No entanto, essa equação deve ser considerada uma abordagem inicial para o monitoramento da capacidade física, ressaltando a importância da repetição da medida em cada consulta do paciente. Estudos futuros podem contribuir para esclarecer a relação entre a variabilidade da FPM e a classificação funcional da IC.

Além disso, embora este estudo tenha incluído um processo de validação interna, a validação externa da equação é essencial para confirmar sua generalização em populações diversas com IC. Essa limitação destaca a necessidade de mais pesquisas para avaliar sua aplicabilidade em diferentes contextos clínicos. Análises adicionais voltadas para a avaliação da utilidade clínica da equação podem favorecer uma adoção mais ampla dessa ferramenta, potencialmente como marcador prognóstico. Isso poderia reduzir a dependência de métodos mais complexos, que frequentemente são difíceis de implementar e serem interpretados em condições clínicas desfavoráveis.

## Conclusão

Encontramos que idade, sexo, estatura, circunferência da panturrilha e classificação NYHA foram os principais determinantes da FPM em indivíduos com IC. A equação preditiva derivada dessas variáveis demonstrou forte concordância com os valores de FPM medidos. Assim, a equação de referência proposta pode servir como uma ferramenta clínica útil para interpretar medições de força de preensão em pacientes com IC.

Disponibilidade de Dados

Os conteúdos subjacentes ao texto da pesquisa estão contidos no manuscrito.

## Material Suplementar

*Material Suplementar Para informação adicional, por favor, clique aqui.
