# Supplementary material for: Força de Preensão Manual na Insuficiência Cardíaca: Construção de uma Equação de Referência
Source: Arq Bras Cardiol. 2025 Oct 13;122(9):e20240777. [Article in Portuguese] doi: 10.36660/abc.20240777 (PMC12674211; doi:10.36660/abc.20240777)

Supplementary material

Supplementary Analysis

Three predictive models were developed:

Model 1: HGS_p = −39.732 + (10.771 * gender [female = 0; male = 1]) - (0.158 * age [years]) + (35.096 * height [m]) - (4.224 * NYHA [I /II = 0; III/IV = 1]) + (0.448 * calf circumference [cm]).

Model 2: HGS_p= −32.037 + (10.406 * gender [female = 0; male = 1]) - (0.170 * age [years]) + (41.335 * height [m]) - (4.300 * NYHA [I /II = 0; III/IV = 1]).

Model 3: HGS_p= −34.498 + (10.598 * gender [female = 0; male = 1]) - (0.188 * age [years]) + (40.396 * height [m]).

Model 1 had an R^2 of 0.578 and an adjusted R^2 of 0.565. When applied to the derivation sample, the mean residual of the observed versus predicted values was 0.025 ± 7.601 kg, which was found to behave symmetrically (P-value = 0.200). Model 2 had an R^2 of 0.561 and an adjusted R^2 of 0.559. In the derivation sample, the mean residual between observed and predicted values was 0.021 ± 7.779 kg, also displaying symmetric behavior (P-value = 0.200). Model 3 showed an R^2 of 0.547 and an adjusted R^2 of 0.544, with a mean residual of 0.013 ± 7.913 kg in the derivation sample, again behaving symmetrically (P-value = 0.200).

The equations were then applied to the validation sample, and Supplementary Figures 1, 2, and 3 illustrate the scatter plots along with their linear fitted lines for models 1, 2, and 3, respectively. The y-axis represents the observed grip strength values (using measurements collected by the dynamometer) of the dominant hand, in kg, while the x-axis represents the predicted values (derived from the reference equations) for the dominant hand.

For Model 1, the Pearson correlation coefficient (r) between the observed and predicted values was 0.69, with an intraclass correlation coefficient of 0.79 (95% CI, 0.69 to 0.86; p < 0.001), indicating good agreement. For Model 2, the Pearson correlation coefficient was 0.68, with an intraclass correlation coefficient of 0.79 (95% CI, 0.68 to 0.86; p < 0.001), also showing good agreement. Model 3 had a Pearson correlation coefficient of 0.68 and an intraclass correlation coefficient of 0.77 (95% CI, 0.66 to 0.84; p < 0.001), demonstrating similar levels of agreement.

In the validation sample, the mean HGS_p according to Model 1 was 29.45 ± 8.90 kg, compared to a mean observed HGS of 30.13 ± 12.33 kg, resulting in a mean residual of -0.68 ± 8.93 kg, meaning the predicted HGS was, on average, 680 g lower than the actual HGS. For Model 2, the mean HGS_p was 29.29 ± 8.85 kg, resulting in a mean residual of 0.78 ± 9.01 kg. For Model 3, the mean HGS_p was 29.44 ± 8.26 kg, with a resulting mean residual of 0.77 ± 9.01 kg.

Supplementary Figure 1. Scatter plot of observed handgrip strength values by model (y-axis) and predicted handgrip strength values 1 (x-axis) in the validation sample. The y-axis represents the observed handgrip strength values (using data from the validation sample) of the dominant hand, in kg, while the x-axis represents the predicted values (derived from the reference equations for heart failure patients by age group) of the dominant hand.

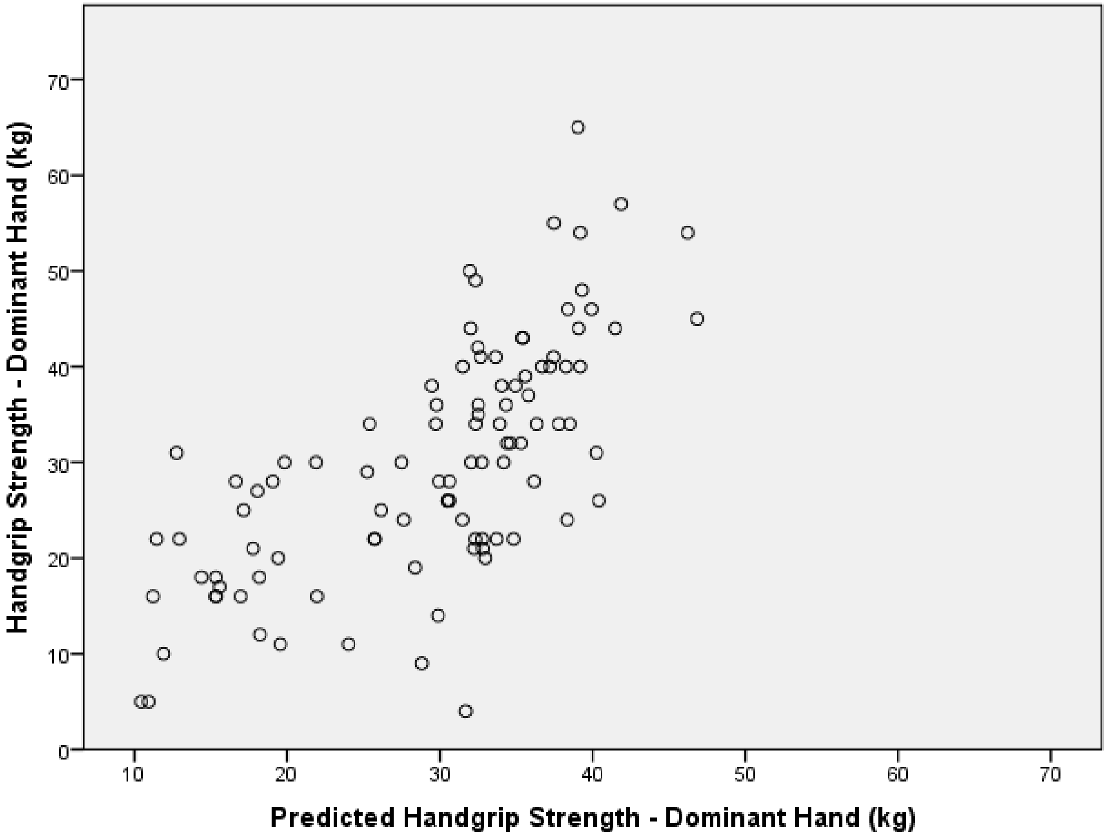

Supplementary Figure 2. Scatter plot of observed handgrip strength values (y-axis) and predicted handgrip strength values by model 2 (x-axis) in the validation sample. The y-axis represents the observed handgrip strength values (using data from the validation sample) of the dominant hand, in kg, while the x-axis represents the predicted values (derived from the reference equations for heart failure patients by age group) of the dominant hand.

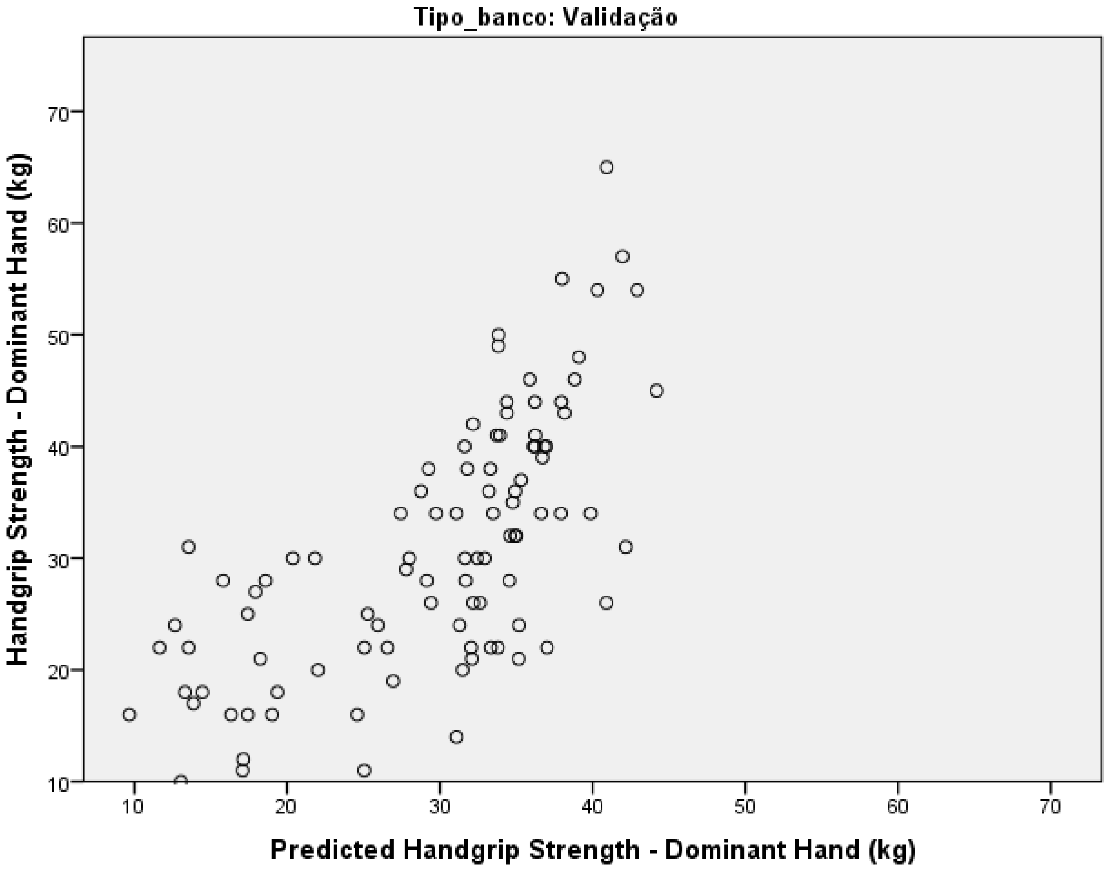

Supplementary Figure 3. Scatter plot of observed handgrip strength values (y-axis) and predicted handgrip strength values by model 3 (x-axis) in the validation sample. The y-axis represents the observed handgrip strength values (using data from the validation sample) of the dominant hand, in kg, while the x-axis represents the predicted values (derived from the reference equations for heart failure patients by age group) of the dominant hand.

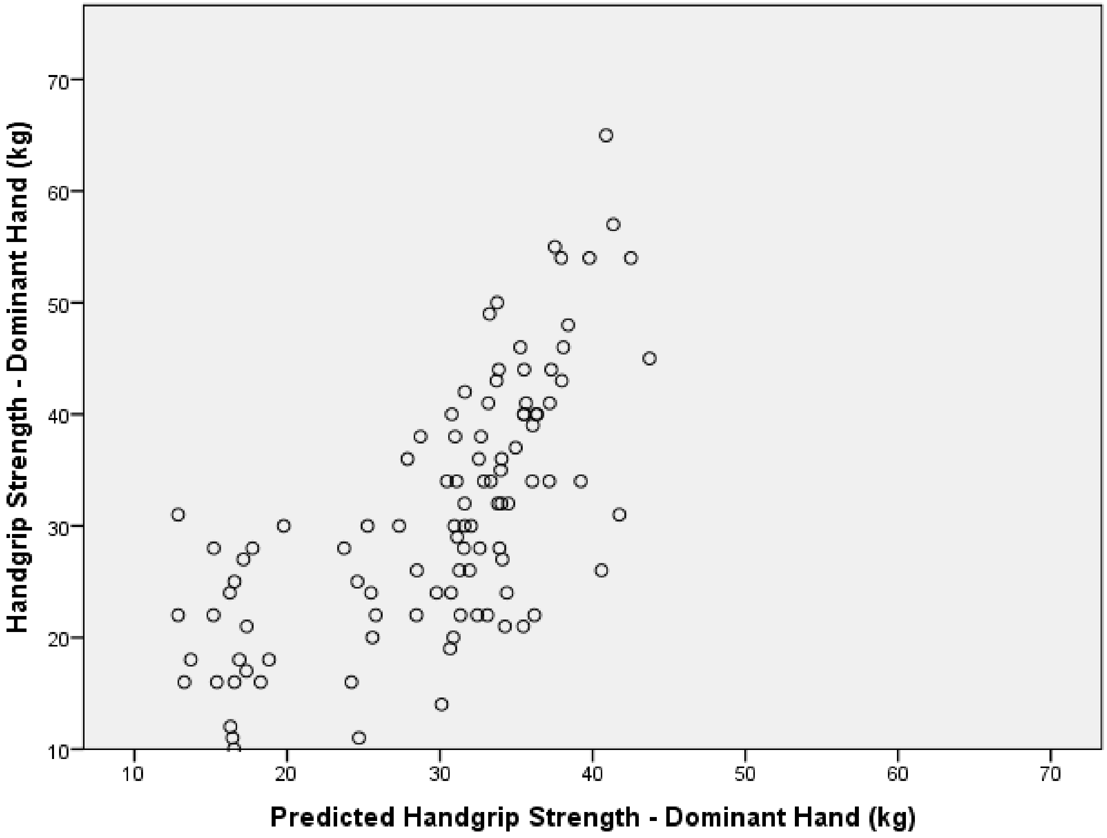

Supplement: *Material Suplementar [file 0066-782X-abc-122-09-e20240777-suppl01.pdf]
