# Supplementary material for: Força de Preensão Manual na Insuficiência Cardíaca: Construção de uma Equação de Referência
Source: Arq Bras Cardiol. 2025 Oct 13;122(9):e20240777. [Article in Portuguese] doi: 10.36660/abc.20240777 (PMC12674211; doi:10.36660/abc.20240777)
Supplement: Translation

# Translation: Handgrip Strength in Heart Failure: Developing a Reference Equation

**DOI:** 10.36660/abc.20240777i

**Authors:** Suena Medeiros Parahiba, Édina Caroline Ternus Ribeiro, Ingrid da Silveira Knobloch, Débowra Dapper, Ingrid Dalira Schweigert Perry, Nadine Oliveira Clausell, Vivian Luft, Gabriela Corrêa Souza, Eneida Rejane Rabelo-Silva

**Affiliations:** 1 Porto Alegre RS Brazil Programa de Pós-Graduação em Ciências da Saúde, Cardiologia e Ciências Cardiovasculares, Faculdade de Medicina, Universidade Federal do Rio Grande do Sul, Porto Alegre, RS – Brazil; 2 Porto Alegre RS Brazil Universidade Federal de Ciências da Saúde de Porto Alegre, Porto Alegre, RS – Brazil; 3 Porto Alegre RS Brazil Hospital de Clínicas de Porto Alegre, Porto Alegre, RS – Brazil; 4 Porto Alegre RS Brazil Programa de Pós-Graduação em Alimentação, Nutrição e Saúde, Faculdade de Medicina, Universidade Federal do Rio Grande do Sul, Porto Alegre, RS – Brazil; 5 Porto Alegre RS Brazil Escola de Enfermagem, Universidade Federal do Rio Grande do Sul, Porto Alegre, RS – Brazil

**Keywords:** Heart Failure, Hand Strength, Reference Values

## Abstract

**Background:**

Handgrip strength (HGS) is a key indicator of overall muscle strength and functional capacity in patients with heart failure (HF). However, no reference equations specific to this population have been previously published.

**Objectives:**

This study aimed to develop and validate a reference equation for predicting HGS in patients with HF.

**Methods:**

A cross-sectional study was conducted on patients with stable HF, aged 18–79 years, diagnosed for at least three months. Maximum HGS value was obtained from three consecutive measurements. Clinical data and anthropometric assessments were collected. The sample was randomly divided into two-thirds (n=174) for derivation and one third (n=100) for validation. A multivariate regression model was applied to develop the predictive equation, including variables with a p-value < 0.25 as determined by the Wald test.

**Results:**

Derivation and validation samples showed no significant differences at baseline. Patients were predominantly male, older adults, and white. The derived equation was: Predicted HGS = −39.732 + (10.771 * gender [female = 0; male = 1]) - (0.158 * age [years]) + (35.096 * height [m]) + (0.448 * calf circumference [cm]) - (4.224 * the New York Heart Association class [I /II = 0; III/IV = 1]). When applied to the validation sample, the equation underestimated actual HGS by 0.68 ± 8.93 Kg.

**Conclusion:**

Age, sex, height, calf circumference, and NYHA class were key determinants of HGS in HF patients. The derived equation showed good predictive accuracy and may serve as a useful reference for interpreting grip strength in this population.

## Introduction

Functional capacity provides a direct and noninvasive assessment of physical status and is associated with prognosis and other clinical outcomes.^1^Handgrip strength (HGS) is known to be associated with frailty, a condition that affects about 40% of HF patients. Frailty is closely linked to ageing, cognitive decline, and increased complexity in clinical management in this population.^2 – 5^

HGS has been investigated as a simple, low-cost alternative for assessing functional capacity, and can be easily applied in clinical settings.^5 , 6^It is a measurement influenced by anthropometric factors, gender, and aging. Although HGS primarily evaluates a group of upper body muscles, it is considered an indicator of overall muscle strength.^7 – 9^

HGS, recently recognized as a prognostic parameter in patients with HF, was evaluated in a systematic review including 7,350 patients with follow-up periods ranging from three to 43 months.^6^It was shown that a 1 Kg decrease in HGS is associated with an 8% increase in mortality risk (relative risk 1.08; 95% confidence interval – CI – 1.05 to 1.11; p < 0.001).

Given the evidence presented, HGS measurement could become a routine marker for assessing and monitoring patients with HF in clinical practice.^5 , 10 , 11^The use of equations and reference tables is an alternative method for predicting and evaluating this parameter. However, existing literature is based on data from healthy populations, and current prediction formulas for HGS are not adapted to clinical conditions that affect hemodynamics, which may lead to inaccurate assessments.^12 , 13^Therefore, this study aimed to develop and validate a reference equation for evaluating HGS in patients with HF. The core methodology of the study is presented in the Central Illustration .

## Methods

### Patient selection and clinical data

This cross-sectional study included adult and elderly patients (18 to 79 years) with a confirmed diagnosis of HF, regardless of their ejection fraction classification. Patients receiving outpatient care were recruited from a HF outpatient clinic of a high-complexity public hospital in Brazil, using a convenience sampling approach. Data were collected from August 2018 to July 2023.

Patients were excluded if they had a history of heart transplant, acutely decompensated HF; acute myocardial infarction, stroke, or surgery within the three months prior to study participation; peripheral congestion and/or edema; history of unstable angina; were undergoing renal replacement therapy; had malignant tumors (active or in remission) within the last five years; acute infection; prior diagnosis of neurodegenerative disease; or were unable to perform functional tests (e.g., wheelchair users, amputees, those with motor sequelae or disabilities). Additional exclusion criteria included contraindications for bioelectrical impedance analysis such as metal prostheses, and body mass index (BMI) >39 Kg/m^2^.

HF etiology, functional classification (according to the New York Heart Association – NYHA), pharmacological treatment, comorbidities, and other clinical and sociodemographic data were obtained from electronic medical records and confirmed during history taking by trained researchers. The data included anthropometric assessment of weight (Kg), height (m), BMI (Kg/m^2^), arm circumference (cm), triceps skinfold (mm), mid-arm circumference (cm), and calf circumference (cm). The assessments were conducted by trained researchers certified by an experienced supervisor prior to data collection.

### Handgrip Strength

HGS was measured using a calibrated Jamar hand dynamometer (Sammons Preston, Bolingbrook, IL, USA). Assessments were performed using the dominant hand, with patients seated, hips flexed at 90°, arms hanging alongside the torso, and elbows bent at 90°.^14 , 15^Patients were instructed to squeeze the handle with maximum effort and received verbal encouragement from the researcher.^16^Three maximal contractions were performed, with a one-minute interval between each attempt. The highest value among the three trials was recorded as the final HGS result.

Predicted HGS (pHGS) was calculated using a reference equation developed for patients with HF, as detailed in the Statistical Analysis section.

### Ethical aspects

This study was approved by a Research Ethics Committee (protocol number 5057.8121.200005327), and all patients signed an informed consent form.

### Statistical analysis

Statistical analysis was performed using SPSS (Statistical Package for the Social Sciences) Version 18.0 (IBM Corp., Armonk, NY, USA). Continuous variables were expressed as mean and standard deviation or median and interquartile range, and categorical variables were expressed as absolute and relative values. Continuous variables were assessed for normality using the Kolmogorov-Smirnov test.

After data collection, two-thirds of the participants were randomly allocated to form a derivation sample using the case selection command in SPSS. The remaining participants were included in the validation cohort. To assess the similarity between the derivation and validation samples, comparisons were made using Pearson's chi-square test ( *χ* ^2^) for categorical variables and the unpaired Student's t-test or the Mann-Whitney U test for continuous variables, as appropriate. A significance level of p = 0.05 was adopted for all comparisons.

To develop the prediction equation in the derivation sample, an initial univariate regression analysis was performed. Variables with a p-value < 0.25 (Wald test) were selected for inclusion in the subsequent multivariate regression model and assessment of the correlation coefficient.^17 – 19^

Three predictive models were developed to estimate HGS in patients with HF (Supplementary Material). The final model was chosen based on its statistical performance and relevance to the characteristics of the studied population. Subsequently, the equation was applied to the validation sample to calculate the pHGS. Predictive accuracy was assessed using residuals and correlation analysis. Residuals were calculated by subtracting the predicted values from the observed values. That is, residuals = observed HGS (measured by dynamometry) - pHGS (derived from the reference equation).

Agreement between observed and predicted values was evaluated using the intraclass correlation coefficient (ICC), which quantifies absolute agreement. ICC values were considered poor if less than 0.5, moderate if between 0.5 and 0.75, good if between 0.75 and less than 0.9, and excellent if between 0.9 and 1.00.^20^Scatter plots were used to visualize the correlation between the observed and predicted values.

## Results

The study included 274 clinically stable HF patients, predominantly older adult men, self-identified as having white skin color, 82% classified as NYHA I or II ( Table 1 ). The derivation sample (n = 174) and the validation sample (n = 100) showed no significant differences in demographic or clinical characteristics.

**Table 1:** Demographic and clinical characteristics of patients with heart failure in the derivation and validation samples by age group

Variable	Derivation Sample (n = 174)	Validation Sample (n = 100)	p-value
Age, years	62.0 (53.8-67)	62.0 (57-67.8)	0.331
Elderly	104 (59.8)	62 (62)	0.716
Male sex	111 (63.8)	74 (74)	0.082
White ethnicity	129 (74.6)	79 (79.0)	0.407
Ejection fraction, %	31 (24-41)	32.5 (25-40)	0.842
NYHA (I/II)	142 (81.6)	84 (84)	0.616
Diabetes	52 (29.9)	31 (31.0)	0.847
SAH	91 (52.3)	48 (48.0)	0.493
CKD	14 (8.0)	11 (11.0)	0.414
Dyslipidemia	17 (9.8)	8 (8.0)	0.624
CAD	13 (7.5)	9 (9.0)	0.654
AF	29 (16.7)	21 (21.0)	0.371
ACE-I/ARB or sacubitril/valsartan	140 (80.0)	82 (81.2)	0.883
Beta blocker	167 (96.5)	96 (96)	0.532
Diuretics	144 (83.2)	87 (87.9)	0.303
Spironolactone	120 (70.6)	68 (69.4)	0.836
Weight, Kg	78.2 (64.5-89.5)	79.3 (65.6-90)	0.930
Height, m	1.6±0.1	1.7±0.1	0.282
BMI, Kg/m²	28.5 (25-31.9)	28.6 (24.3-30.9)	0.722
AC, cm	32.7±4.1	32.4±4.2	0.609
TSF, mm	17.1 (13.1-23.9)	16.1 (12.6-21.7)	0.335
MAC, cm	26.7±2.9	26.8±3.7	0.898
CC, cm	38.1±3.9	37.9±3.7	0.744
HGS, kg	28 (20-36.2)	30 (22-39.3)	0.176

AC: arm circumference; AF: atrial fibrillation; CAD: coronary artery disease; CC: calf circumference; CKD: chronic kidney disease; HF: heart failure; HGS: handgrip strength; pHGS: predicted handgrip strength; MAC: mid-arm circumference; NYHA: congestive heart failure functional class according to New York Heart Association; SAH: systemic arterial hypertension; TSF: triceps skinfold. Continuous variables are expressed as mean and standard deviation or median and interquartile range, and categorical variables are expressed as absolute and relative values.

In the analysis of the derivation sample (n = 174), the variables with the greatest explanatory power for HGS were sex, age, height, calf circumference and NYHA functional class, with adjusted R^2^values of 0.427, 0.043, 0.446, 0.093 and 0.031 respectively. Based on these variables, the prediction equation for HGS in HF patients (18 to 79 years) was: pHGS = −39.732 + (10.771 * gender [female = 0; male = 1]) - (0.158 * age [years]) + (35.096 * height [m]) + (0.448 * calf circumference [cm]) - (4.224 * NYHA [I /II = 0; III/IV = 1]) ( Table 2 ). This model had an R^2^of 0.578 and an adjusted R^2^of 0.565. When applied to the derivation sample itself, the mean residual of the observed versus predicted values was 0.025 ± 7.601 Kg, which was found to be a symmetrically behaving variable (p-value = 0.200).

**Table 2:** Coefficients and statistics of the multivariate regression analysis for handgrip strength (dominant hand) in the derivation sample of patients with heart failure aged 18 to 79 years

Model	Unstandardized Coefficient, B	95% CI	p-value
Constant	-39.732	-70.070, -9.394	0.01
Sex (female = 0; male = 1)	10.771	7.308, 14.233	<0.001
Age (years)	-0.158	-0.261, -0.054	0.003
Height (m)	35.096	17.251, 52.941	0.000
Calf circumference (cm)	0.448	0.138, 0.758	0.005
NYHA (I/II = 0; III/IV = 1)	-4.224	-7.234, -1.213	0.006

CI: confidence interval; HF: heart failure; HGS: handgrip strength; NYHA: congestive heart failure functional class according to New York Heart Association.

### Correlation

The equation was applied to the validation sample. The scatter plots and their linear fitted lines are shown in Supplementary Figure 1. The y-axis represents the observed grip strength values (measured with a dynamometer) of the dominant hand, and the x-axis shows the predicted values (in Kg), derived from the reference equation, for the dominant hand. Overall, the Pearson correlation coefficient (r) between the observed and predicted values was 0.69, and the ICC was 0.79 (95% CI, 0.69 to 0.86; p < 0.001), demonstrating good agreement.

### Residuals

In the validation sample, the mean pHGS was 29.45 ± 8.90 Kg, compared to a mean observed HGS of 30.13 ± 12.33 Kg. This resulted in a mean residual of 0.68 ± 8.93 Kg, indicating that the pHGS was, on average, 680 g lower than the observed value.

## Discussion

This study proposes a reference equation for predicting HGS values in patients with HF, based on data collected from clinically stable individuals. The data were obtained following protocols published in the literature.^14 , 15 , 17 – 19 , 21^The results indicate that 57% of the variance in HGS can be explained by the combined influence of gender, age, height, NYHA classification, and calf circumference. The proposed equation provides an alternative for estimating expected HGS values in HF, allowing clinicians and researchers to compare measurements in outpatients and potentially extrapolate its use to decompensation scenarios.

Despite the strong correlation found, the residuals between predicted and observed HGS values cannot be overlooked. The same has been described in a population-based study involving healthy individuals, where HGS was predicted based on age, height, and weight.^22^In that study, the mean residuals were 1.41 ± 5.57 Kg and 1.03 ± 5.44 kg for the dominant and non-dominant hands, respectively.^22^Wang et al. also shown that pHGS values may differ from measured HGS in a considerable proportion of patients.^22^

In our previous literature review, no reference equations were identified that specifically apply to a population with HF. However, Brazilian research groups have developed reference equations for healthy individuals from the general Brazilian population.^23 , 24^For example, Novaes et al.^23^proposed the following equations: Dominant hand HGS = 39.996 − (0.382 × age [years]) + (0.174 × weight [Kg]) + (13.628 × sex [female = 0; male = 1]); non-dominant hand HGS = 44.968 − (0.420 × age [years]) + (0.110 × weight [kg]) + (9.274 × sex [female = 0; male = 1]).^23^

This study included only individuals aged 50 years or older and reported adjusted regression coefficients of 0.677 for the dominant hand and 0.546 for the non-dominant hand.^23^However, when tested in a population of middle-aged Brazilian men, the ICCs between the predicted and measured values were low (0.52 for the dominant hand and 0.42 for the non-dominant hand), indicating limited predictive accuracy.^25^These results suggest that the equations may have limited validity when extrapolated to populations beyond those originally studied.

In contrast, Lopes et al.^24^developed predictive equations for HGS in healthy young adult and middle-aged Brazilian individuals, incorporating additional variables such as forearm circumference and hand length, in addition to gender. The proposed equations were: Dominant hand HGS = −15.490 + (10.787 × gender [female = 0; male = 1]) + (0.558 × forearm circumference, [cm]) + (1.763 × hand Length [cm]); Non-dominant had HGS = - 9.887 + (12.832 × gender [female = 0; male = 1]) + (2.028 × hand length [cm]). These equations had the capacity to explain 71% of the variability in HGS.^24^

An analysis of the reference equations reveals that certain variables have a more significant influence on HGS. One study showed that, among the anthropometric measurements of the forearm and hand, hand width is the best predictor of HGS in young adults.^26^Nevertheless, height is widely recognized in the literature as the anthropometric variable most closely associated with HGS. Height reflects part of bone structure, and bone mass also contributes to muscle and strength and performance.^9 , 27 – 29^The International Working Group on Sarcopenia recommends including height when assessing relative muscle mass, particularly in the context of functional disability.^30^Thus, incorporating height into predictive models for HGS is essential to obtain values that are consistent with an individual's structural and anatomical characteristics.

As expected, there was a difference in strength between men and women, which extended to other upper-limb muscle groups. Women exhibit approximately 30% of male strength in shoulder flexion and internal/external rotation. Although the scapular strength mechanism remains consistent across movement patterns, women generate only 55% to 62% of the strength observed in men.^31^In terms of wrist strength, women produce approximately 60–65% of male flexion/extension strength, about 55-60% of pronation/supination strength, and 60–70% of ulnar/radial deviation force.^32^Overall, women exhibit lower force production capacities compared to men; however, when strength is normalized by body mass, the gender differences become less pronounced, and in some cases, women surpass men in strength metrics. This phenomenon is particularly evident during flexion and extension movements under isokinetic conditions.^32^

Age also plays a significant role in predicting an individual's HGS, primarily due to age-related declines in muscle strength.^8^In humans, both the neuromuscular and sensory systems are essential to generating maximum grip force.^33 , 34^Over time, physiological degradation in these systems can lead to slower and less consistent grip strength responses.

The prediction equation for individuals with HF required the inclusion of calf circumference, an indicator of lean body mass. This decision is supported by previous research indicating that HGS correlates with overall muscle strength in patients.^7^Furthermore, the inclusion of the NYHA functional classification introduces a widely used clinical variable, based on the assessment of physical activity limitations.^35^A previous study demonstrated clinically significant differences in HGS among patients with greater functional impairment, as classified by NYHA III/IV. Specifically, for each additional kilogram in HGS, there was a 2% decrease in the likelihood of being classified as NYHA III or IV.^36^

Condition-specific HGS prediction equations can serve as valuable tools for clinical interpretation, as patients often exhibit baseline values significantly below the reference standards established for healthy populations. To address the limitations posed by the absence of specific reference values for patients undergoing hemodialysis, Dilloway et al.^37^proposed a predictive equation tailored to this group. The derivation sample comprised well-nourished individuals, as assessed by the Subjective Global Assessment. Separate equations were developed for each gender using demographic variables such as height and age. Using these equations, the expected HGS was calculated and compared to the observed HGS measured by dynamometry. This comparison yielded the HGS index (%), which the authors proposed as personalized metric offering a more precise assessment of muscle weakness in patients undergoing hemodialysis.^37^

To our knowledge, this is the first study to propose a prediction equation for HGS in patients with HF. Although this study presents limitations, such as reliance on a convenience sample and the lack of longitudinal HGS monitoring within the same individuals, the strong correlations and high predictive accuracy of the equation suggest it could become a valuable tool in clinical practice, particularly for assessing frailty. Nevertheless, this equation should be viewed as an initial approach to monitoring physical capacity, highlighting the importance of repeated measurements at each patient visit. Future studies may help elucidate the relationship between HGS variability and HF functional classification.

Additionally, although this study included an internal validation process, external validation of the equation is essential to confirm its generalizability across diverse HF populations. This limitation highlights the need for further research to evaluate its applicability in different clinical settings. Further analyses aimed at evaluating the clinical utility of the equation could encourage broader adoption of this tool, potentially as a prognostic marker.^6^This could reduce reliance on more complex methods that are often difficult to implement and interpret in unfavorable clinical conditions.

## Conclusion

We found that age, sex, height, calf circumference, and NYHA classification were key determinants of HGS in individuals with HF. The predictive equation derived from these variables demonstrated strong concordance with measured HGS values. As such, the proposed reference equation can serve as a useful clinical tool for interpreting grip strength measurements in patients with HF.

Data Availability

The underlying content of the research text is contained within the manuscript.

## Supplementary Materials

*Supplemental Materials For additional information, please click here.
